# Nasal mucociliary transportability of male and female smokers^☆^

**DOI:** 10.1016/j.bjorl.2017.03.006

**Published:** 2017-04-08

**Authors:** Juliana Souza Uzeloto, Dionei Ramos, Ana Paula C.F. Freire, Diego G.D. Christofaro, Ercy Mara C. Ramos

**Affiliations:** Universidade Estadual Paulista (UNESP), Faculdade de Ciências e Tecnologia, São Paulo, SP, Brazil

**Keywords:** Mucociliary transport, Mucociliary clearance, Sex characteristics, Tobacco use disorder, Smoking, Saccharin, Transporte mucociliar, Clearance mucociliar, Características sexuais, Distúrbio do tabagismo, Fumo, Sacarina

## Abstract

**Introduction:**

Female smoker's present increased susceptibility to several diseases when compared to the opposite gender. However, there are no studies showing differences in nasal mucociliary transport behavior between male and female smokers.

**Objective:**

To compare the nasal mucociliary transportability in male and female smokers and non-smokers, taking into consideration age, anthropometric data, smoking load and pulmonary function.

**Methods:**

The analysis included 139 individuals (33 men and 37 women smokers and 32 men and 37 women non-smokers). All participants answered an initial interview to obtain personal data and smoking load. Anthropometric data and carbon monoxide in the exhaled air were assessed. Individuals also performed pulmonary function test and Saccharin Transit Time test. To compare saccharin transit time values between men and women, smokers and non-smokers, stratification of all independent variables was performed (sociodemographic, smoking and respiratory variables) into two categories: below and above the median values.

**Results:**

There was no difference between men and women, smokers and non-smokers, regarding nasal mucociliary transportability. Significant differences were only observed between non-smokers. Among those with less forced vital capacity values (<97.37% of predicted), women presented mucociliary transport faster than men. Moreover, it was observed influence of BMI and COex (women smokers), FCV and FEV1 (men non-smokers) and FEF_25–75%_ (women non-smokers) on saccharin transit time values.

**Conclusion:**

Based on the findings of this study, nasal mucociliary transport in male and female adult smokers, apparently healthy, are similar.

## Resumo

**Introdução:**

Mulheres tabagistas apresentam maior susceptibilidade à diversas doenças quando comparadas ao sexo masculino. No entanto, não há estudos mostrando diferenças no comportamento do transporte mucociliar nasal entre tabagistas do sexo masculino e feminino.

**Objetivo:**

Comparar a transportabilidade mucociliar nasal em homens e mulheres fumantes e não fumantes, levando em consideração idade, dados antropométricos, carga tabágica e função pulmonar.

**Método:**

A análise incluiu 139 indivíduos (33 homens e 37 mulheres fumantes e 32 homens e 37 mulheres não fumantes). Todos os participantes responderam a uma entrevista inicial para a obtenção de dados pessoais e a carga tabágica. Dados antropométricos e monóxido de carbono no ar expirado foram avaliados. Os indivíduos também realizaram teste de função pulmonar e o teste de trânsito de sacarina. Para comparar os valores do teste de trânsito de sacarina entre homens e mulheres, fumantes e não fumantes, foi realizada a estratificação de todas as variáveis independentes (variáveis sociodemográficas, tabágicas e respiratórias) em duas categorias: abaixo e acima dos valores médios.

**Resultados:**

Não houve diferenças entre homens e mulheres, fumantes e não fumantes, em relação à transportabilidade mucociliar nasal. Diferenças significativas foram observadas apenas entre não-fumantes. Entre os que apresentaram valores menores de capacidade vital forçada (<97,37% do previsto), as mulheres apresentaram transporte mucociliar mais rápido que os homens. Além disso, observou-se influência do IMC e COex (mulheres fumantes), CVF e VEF_1_ (homens não fumantes) e FEF_25%-75%_ (mulheres não fumantes) sobre os valores do teste de trânsito de sacarina.

**Conclusão:**

Com base nos achados desse estudo, o transporte mucociliar nasal em tabagistas masculinos e femininos adultos, aparentemente saudáveis, são semelhantes.

## Introduction

Female smokers present greater susceptibility to various diseases when compared to the opposite gender, being more likely to develop cancer, ischemic heart disease, and Chronic Obstructive Pulmonary Disease (COPD). Women are more prone to develop COPD quicker, with lower smoking exposure, and greater severity, showing an increased mortality rate.^1^

Langhammer et al.^2^ reported that female smokers present narrowing of the airways and increased bronchial hyper responsiveness, with superior intensity than men. This can be explained by hormonal levels, whereby estrogen increases the bioactivation of many compounds in tobacco.^3^, ^4^ This finding is disturbing considering that estimations of future generations will have approximately 500 million female smokers.^5^

Regarding nasal mucociliary transport, the main mechanism of defense of the respiratory system, there is no evidence showing differences between male and female smokers. However, studies performed on non-smokers show some differences; some report that women exhibit better transportability than men,^6^, ^7^, ^8^ while others^9^, ^10^ report no relationship between sex and mucociliary transportability. Furthermore, it is necessary to consider some variables that could possibly interfere in the analysis of this relationship, such as sociodemographic, anthropometric and lifestyle.

Given the above, it is necessary to evaluate the influence of gender on nasal mucociliary transportability in smokers, to make campaigns and programs against tobacco use more intense and specific to the population that presents the greatest harm.

Thus, the aim of this study was to compare nasal mucociliary transportability in male and female smokers and non-smokers, taking into consideration age, anthropometric data, smoking load, and pulmonary function.

## Methods

This study has a cross-sectional design and the sample is composed of smokers, recruited according to the availability of individuals participating in the University smoking cessation group. All participants were informed about the purpose and procedures of the study and signed a consent form.

The project was approved by the Ethics Committee of the proponent Institution of the study (Protocol no. 18/2011).

Inclusion criteria was defined as: age between 30 and 50 years old; both genders; normal lung function (assessed by spirometry); no diagnosis of pulmonary disease; no history of surgery or nasal trauma, nasal septum deviation, infection or inflammation in the respiratory system observed in clinical evaluation during the interview and experimental protocol; and smoking for at least one year (smoker group). Subjects who did not understand or cooperate with the procedures of the study or failure to attend assessments days were excluded.

The assessments were performed over two days. The first day included interviews to obtain personal data, anthropometric measurements, questions about smoking habits and dependence on nicotine and assessment of pulmonary function. The second day included measurements of carbon monoxide level in exhaled air and nasal mucociliary transportability. All assessments were performed during the morning.

During both days, the volunteers were instructed not to use alcohol, caffeine based substances, drugs (such as anesthetics, painkillers, barbiturates, tranquilizers and antidepressants), and not to smoke during the 12 h prior to the assessments, because these substances alter nasal mucociliary transportability and bronchi dilation.^11^, ^12^

### Initial assessment

After inclusion in the study, all participants provided information about: date of birth, education level, history of smoking (cigarettes smoked per day, number of years smoking; from this the packs/year index was calculated, which is the number of cigarettes smoked per day, divided by 20 and multiplied by the number of years the individual smoked),^13^ and clinical history of respiratory events (surgery or nasal trauma, chronic respiratory diseases, infections in the last weeks). Weight and height were determined using a scale and stadiometer (Sanny^®^, Brazil) then Body Mass Index (BMI) was calculated. This data was collected during the first day of assessment.

### Nicotine dependence

The level of nicotine dependence was assessed using the Fagerström Nicotine Dependence Questionnaire.^14^ The instrument consists of a questionnaire with six easy to understand items. The scores obtained in the test allow the classification of nicotine dependence into five levels: very low (0–2 points); low (3–4 points); moderate (5 points); high (6–7 points); and very high (8–10 points).^15^ This assessment was performed during the first day.

### Pulmonary function (spirometry)

For measurement of pulmonary function, spirometry was performed using a spirometer (MIR-Spirobank version 3.6) coupled to a computer, according to the guidelines and criteria established by the European Respiratory Society.^16^ The normality values were from the Brazilian population.^17^ This assessment was performed on all participants during the first day.

### Carbon monoxide in exhaled air (COex)

The measurement of COex was used to verify the 12 h tobacco abstinence period. The application of this technique was standardized as described: the volunteer was instructed to inhale deeply and then remain in apnea for 20 s. After that, the device (Micro Medical Ltda., Rochester, Kent, UK) was placed in the mouth of the individual and they were asked to perform a full exhalation, slowly and smoothly.^18^ This assessment was performed during the second day.

### Nasal mucociliary transport (saccharin transit time test)

Evaluation of mucociliary transport was performed using the STT. The test was conducted at a room temperature of 25 °C and relative humidity between 50% and 60%. The participants were seated with their head extended to 10. The STT was initiated by introducing approximately 2.5 mg of granulated saccharin sodium through a plastic straw, under visual control, approximately 2 cm into the right nostril. From this moment, the timer was activated and the individuals were advised not to: walk, talk, cough, sneeze, scratch or blow their nose, and were instructed to swallow their saliva a few times per minute until they felt some taste in their throat; when the participant alerted the examiner with a gesture, the time was recorded.^19^ All volunteers performed this evaluation during the second day.

## Statistical analysis

The final sample was calculated considering a two-tailed hypothesis test that used an equation based on a confidence level of 5%, 80% test power and standard deviation of 4.1 (based on a previous study),^20^ which indicated the need for 33 volunteers per group. The normal distribution of data was assessed using the Kolmogorov–Smirnov test and because normality was not detected, Mann–Whitney test was used to compare data between men and women. To compare STT values between men and women, stratification was carried out on all independent variables (sociodemographic, smoking and respiratory variables) into two categories: below and above the median values. Data are expressed as median and interquartile range. Linear multiple regression analysis assessed the influence of the multiple variables (age, BMI, education, smoking years, cigarettes per day, pack/years, nicotine dependence, COex and respiratory variables) on STT values. All statistical analyses were performed by the software SPSS (release 17.0) and statistical significance (*p*-value) was set at 0.05.

## Results

A total of 139 subjects were analyzed and divided into four groups according to gender and smoking habit: 70 smokers (33 men and 37 women) and 69 non-smokers (32 men and 37 women).

The characteristics of the groups are shown in Table 1 There were no significant differences for age, BMI or education level between male and female smokers and non-smokers. Regarding smoking habits, values were similar for number of years smoking, number of cigarettes per day and the packs/year among smokers. The scores of the Fagerström questionnaire were similar between the smoking groups and indicated high nicotine dependence. Carbon monoxide level in exhaled air was significantly higher among smokers.

**Table 1:** Characterization of the sample by groups. The data are expressed in median and interquartile range.

Variables	Smokers		Non-smokers		*p*-value
	Men (*n* = 33)	Women (*n* = 37)	Men (*n* = 32)	Women (*n* = 37)	
Age (years)	42.00 (15.00)	40.00 (10.00)	40.00 (11.00)	39.00 (9.00)	0.99
Weight (kg)	84.00 (21.75)	65.20 (21.40)	82.00 (12.58)	64.60 (17.00)	<0.0001^a^
Height (m)	1.74 (0.09)	1.63 (0.01)	1.74 (0.10)	1.62 (0.09)	<0.0001^a^
BMI (kg/m^2^)	27.17 (6.22)	25.48 (5.85)	27.17 (4.00)	25.10 (4.40)	0.07
Education (years)	11.00 (7.00)	11.00 (3.00)	11.00 (7.00)	11.00 (2.25)	0.17
Years of smoking	20.00 (14.00)	23.00 (12.00)	–	–	0.89
Cigarettes per day	20.00 (22.00)	20.00 (9.00)	–	–	0.06
Pack/years	24.00 (29.40)	20.00 (15.38)	–	–	0.36
Nicotine dependence	7.00 (4.00)	7.00 (3.00)	–	–	0.95
COex (%Hb)	1.60 (1.36)	0.96 (0.96)	0.16 (0.48)	0.16 (0.48)	<0.0001^a^

Hb, hemoglobin; BMI, body mass index.

a *p* < 0.05.

Table 2 presents the median values of STT according to age, BMI, education level, years smoking, number of cigarettes per day, packs/year, nicotine dependence, and Carbon monoxide values in exhaled air (COex). It was observed that, even considering sociodemographic, smoking variables (number of years smoking, number of cigarettes per day, packs/year, and nicotine dependence) and carbon monoxide levels, there was no significant difference when comparing STT in male and female smokers and non-smokers (*p* > 0.05).

**Table 2:** Comparison of saccharin transit time test (minutes) values between male and female smokers and non-smokers according to sociodemographic and smoking variables, and carbon monoxide in exhaled air. The data are expressed in median and interquartile range.

Variables		Smokers		Non-smokers		*p*-value
		Men (*n* = 33)	Women (*n* = 37)	Men (*n* = 32)	Women (*n* = 37)	
Age (years)	<41	10.55 (7.31)	10.09 (8.12)	7.03 (15.23)	8.05 (6.69)	0.33
	≥41	10.23 (9.00)	7.93 (6.03)	10.83 (10.27)	10.67 (11.69)	0.35
BMI (kg/m^2^)	<25.87	10.37 (7.22)	11.4 (8.78)	6.73 (14.21)	8.58 (8.20)	0.32
	≥25.87	9.76 (8.41)	7.93 (4.62)	10.83 (13.31)	9.13 (7.25)	0.12
Education (years)	<11	12.30 (13.03)	10.79 (9.86)	8.53 (6.61)	9.51 (7.09)	0.86
	≥11	9.86 (5.91)	8.05 (7.05)	8.58 (15.26)	6.83 (8.16)	0.35
Smoking years	<21	10.28 (5.97)	8.00 (9.61)	–	–	0.08
	≥21	10.55 (7.31)	9.22 (9.00)	–	–	0.54
Cigarettes per day	<20	10.30 (3.54)	10.78 (10.32)	–	–	0.73
	≥20	9.61 (5.85)	10.30 (10.22)	–	–	0.30
Pack/years	<20.87	10.33 (8.73)	9.85 (8.87)	–	–	0.50
	≥20.87	9.84 (8.29)	9.09 (4.44)	–	–	0.64
Nicotine dependence (points)	<7	10.23 (6.62)	11.15 (10.35)	–	–	0.74
	≥7	10.87 (11.78)	8.63 (4.95)	–	–	0.19
COex (%Hb)	<1.12	10.55 (11.74)	11.14 (6.38)	8.58 (11.67)	8.59 (6.84)	0.46
	≥1.12	10.30 (4.95)	8.63 (5.08)	–	3.55 (0.00)	0.22

Hb, hemoglobin; BMI, body mass index.

Table 3 shows variables of pulmonary function. Even considering the values according to the median for each variable, a significant difference was observed between non-smokers. Among those with less forced vital capacity values (<97.37% of predicted), women presented mucociliary transport faster than men.

**Table 3:** Comparison of saccharin transit time test (minutes) values between male and female smokers and non-smokers according to pulmonary function variables. The data are expressed in median and interquartile range.

Variables		Smokers		Non-smokers		*p*-value
		Men (*n* = 33)	Women (*n* = 37)	Men (*n* = 32)	Women (*n* = 37)	
FVC (% of predicted)	<97.37	10.37 (8.49)	13.16 (9.31)	15.46 (14.84)	6.17 (5.54)	0.0352^a^
	≥97.37	8.73 (6.12)	8.63 (5.03)	7.05 (5.99)	9.45 (9.24)	0.7544
FEV_1_ (% of predicted)	<93.40	10.59 (10.33)	11.8 (9.53)	15.57 (21.41)	8.6 (8.55)	0.0613
	≥93.40	9.48 (5.38)	9.55 (4.80)	7.05 (5.89)	8.32 (7.78)	0.7543
FEV_1_/FVC (%)	<80.55	8.25 (10.00)	8.28 (6.21)	9.25 (8.53)	8.6 (8.22)	0.6892
	≥80.55	10.55 (6.77)	10.78 (6.45)	7.9 (17.44)	8.3 (9.99)	0.5509
PEF (% of predicted)	<96.25	10.3 (11.16)	10.78 (8.17)	11.49 (15.59)	7.44 (8.06)	0.2173
	≥96.25	10.3 (7.24)	9.09 (5.74)	7.05 (10.47)	8.67 (7.61)	0.9521
FEF_25–75%_ (% of predicted)	<89.10	8.11 (7.16)	6.05 (9.00)	10.55 (10.64)	4.82 (6.71)	0.0981
	≥89.10	11.38 (6.69)	10.56 (5.49)	7.47 (16.29)	9.45 (9.41)	0.5813

FVC, forced vital capacity; FEV_1_, forced expiratory volume in one second; PEF, peak expiratory flow; FEF_25–75%_, forced expiratory flow between 25% and 75%.

a *p* < 0.05.

Regression analyses showed influence of BMI and COex (women smokers), FCV and FEV_1_ (men non-smokers) and FEF_25–75%_ (women non-smokers) on STT values (Table 4).

**Table 4:** Multiple regression analysis between multiple variables (age, body mass index, education, smoking years, cigarettes per day, pack/years, nicotine dependence, carbon monoxide in exhaled air and respiratory variables) and saccharin transit time test (minutes).

	Smokers								Non-smokers							
	Men (*n* = 33)				Women (*n* = 37)				Men (*n* = 32)				Women (*n* = 37)			
	*β*	95% CI		*p*	*β*	95% CI		*p*	*β*	95% CI		*p*	*β*	95% CI		*p*
		Lower limit	Upper limit			Lower limit	Upper limit			Lower limit	Upper limit			Lower limit	Upper limit	
Age	0.07	−0.22	0.36	0.63	−0.01	−0.28	0.26	0.94	0.41	−0.17	0.99	0.16	0.07	−0.31	0.45	0.73
BMI	0.03	−0.48	0.43	0.90	−0.40	−0.74	−0.06	0.02^a^	0.38	−0.64	1.40	0.45	−0.00	−0.41	0.40	0.98
Education	0.20	−0.69	0.29	0.41	−0.29	−0.64	0.05	0.09	0.11	−0.97	1.18	0.84	−0.63	−1.29	0.03	0.06
Smoking years	0.02	−0.24	0.28	0.87	0.16	−0.05	0.37	0.14	–	–	–	–	–	–	–	–
Cigarettes per day	0.07	−0.10	0.23	0.40	−0.02	−0.17	0.12	0.74	–	–	–	–	–	–	–	–
Pack/years	0.03	−0.08	0.14	0.57	0.01	−0.12	0.15	0.85	–	–	–	–	–	–	–	–
Nicotine dependence	0.20	−0.61	1.02	0.61	−0.27	−1.02	0.47	0.46	–	–	–	–	–	–	–	–
COex (% Hb)	1.18	−3.6	1.26	0.33	−2.47	−4.75	−0.19	0.04^a^	4.40	−8.47	17.27	0.49	−1.03	−6.80	4.75	0.72
FVC (% of predicted)	0.14	−0.38	0.10	0.25	−0.04	−0.18	0.11	0.59	−0.29	−0.50	−0.09	0.01^a^	0.08	−0.07	0.23	0.30
FEV_1_ (% of predicted)	0.11	−0.35	0.13	0.37	0.00	−0.14	0.15	0.98	−0.31	−0.55	−0.08	0.01^a^	0.13	−0.02	0.28	0.09
FEV_1_/FVC	0.06	−0.31	0.43	0.76	0.25	−0.13	0.63	0.19	0.28	−0.29	0.84	0.32	0.42	−0.05	0.88	0.08
PEF (% of predicted)	0.09	−0.23	0.05	0.21	−0.06	−0.16	0.05	0.27	−0.14	−0.37	0.08	0.20	0.08	−0.04	0.20	0.18
FEF_25–75%_ (% of predicted)	0.01	−0.11	0.12	0.92	0.03	−0.04	0.10	0.37	0.01	−0.13	0.15	0.87	0.09	0.01	0.16	0.03^a^

Hb, hemoglobin; FVC, forced vital capacity; FEV_1_, forced expiratory volume in one second; PEF, peak expiratory flow; FEF_25–75%_, forced expiratory flow between 25% and 75%.

a *p* < 0.05.

## Discussion

The findings of this study showed that gender did not influence the main mechanism of defense of the respiratory system, the mucociliary transport, showing similar responses in male and female smokers, even after stratification for age, BMI, education level, smoking load and lung function.

Several studies with smokers have analyzed the influence of gender in different variables and systems (anxiety,^21^ stress,^22^, ^23^ sarcoidosis,^24^ lung cancer,^25^ oral cancer,^26^ and COPD,^27^ among others). However, this is the first study to analyze the influence of gender on mucociliary transportability in smokers.

Studies involving healthy individuals, non-smokers, have been carried out to investigate the influence of gender on mucociliary transportability, but there are disagreements in the findings. Oliveira-Maul et al.^10^ analyzed nasal mucociliary transportability of 79 individuals, aged between 18 and 94 years old, non-smokers, and observed no significant differences in the relationship between transportability and gender; the researchers used STT to evaluate the mucociliary transport, similar to the present study. However, it was observed in other studies^6^, ^7^, ^8^ that women, and non-smokers, presented better mucociliary transportability when compared to men. In the present study, the only difference observed regarding transportability in non-smokers with forced vital capacity less than 97.37% of predicted. Women presented lower values of STT than men, corroborating with results found by Armengot et al.,^6^ Gerrard et al.^7^ and Bennett et al.^8^ One possible explanation for this difference is that women may have shorter proximal bronchi^28^ and, consequently, shorter transit times and particle clearance.

One possible explanation for the lack of differences in the present study is the young age of the population studied (30–50 years old). Even being classified as moderate smokers^29^ (20 cigarettes per day in both groups), the mucociliary transport of the participants may not be adversely affected yet, as the STT of non-smokers in the literature (8 min), with a similar average age^29^ presents no discrepancy with the values of smokers in the present study.

Several external factors may influence mucociliary transport.^11^ During the present study, it was assessed the effect of internal variables. Among women smokers, higher values of BMI and COex may negatively influence STT. Among non-smokers, the respiratory variables may influence STT, in men the FEF_25–75%_ index and in women the FVC and FEV_1_ values.

Knowing that female smokers are more vulnerable to develop COPD when compared to men^1^ and considering the findings of this study, we suggest that future studies, with older populations, investigate whether there are differences in the transportability between men and women in different age groups, since women may suffer more intense harm from smoking.

One factor that cannot be dismissed is the influence of gender hormones on mucociliary transportability. Jain et al.,^30^ suggested that progesterone receptors in the airways have an important role in inhibiting the ciliary beat, and women have a slower ciliary beat than men due to differences in progesterone concentration. On the other hand, there is different concentrations of gender hormones during different periods of the menstrual cycle, so mucociliary transport is dependent on the menstrual period.^31^ Thus, this limitation should be addressed because this factor was not evaluated. Another limitation is the study is the cross-sectional design, which does not allow the analysis of causal relationships. However, positive aspects of this study, we highlight the originality of the investigation (influence of gender on nasal mucociliary transportability in smokers), which has not been investigated previously, moreover, we also verified transportability was different between genders considering confounding factors, such as sociodemographic characteristics, smoking load, and lung function.

## Conclusion

Nasal mucociliary transport in male and female adult smokers (with no previous health conditions related to smoking) is similar.

## Funding

Research Support Foundation of the State of São Paulo (Process no. 2014/11970-3) (Opinions, hypotheses, and conclusions or recommendations expressed in this material are those of the authors and do not necessarily reflect the views of FAPESP).

## Conflicts of interest

The authors declare no conflicts of interest.
